# Natural Plant Products: A Less Focused Aspect for the COVID-19 Viral Outbreak

**DOI:** 10.3389/fpls.2020.568890

**Published:** 2020-10-15

**Authors:** Anamika Pandey, Mohd Kamran Khan, Mehmet Hamurcu, Sait Gezgin

**Affiliations:** Department of Soil Science and Plant Nutrition, Faculty of Agriculture, Selcuk University, Konya, Turkey

**Keywords:** clinical trials, coronavirus, coronavirus disease of 2019, natural compounds, molecular docking, phytoconstituents, severe acute respiratory syndrome coronavirus 2

## Abstract

The sudden emergence of COVID-19 caused by a novel coronavirus (nCoV) led the entire world to search for relevant solutions to fight the pandemic. Although continuous trials are being conducted to develop precise vaccines and therapeutic antibodies, a potential remedy is yet to be developed. Plants have largely contributed to the treatment of several human diseases and different phytoconstituents have been previously described to impede the replication of numerous viruses. Despite the previous positive reports of plant-based medications, no successful clinical trials of phyto-anti-COVID drugs could be conducted to date. In this article, we discuss varying perspectives on why phyto-anti-viral drug clinical trials were not successful in the case of COVID-19. The issue has been discussed in light of the usage of plant-based therapeutics in previous coronavirus outbreaks. Through this article, we aim to identify the disadvantages in this research area and suggest some measures to ensure that phytoconstituents can efficiently contribute to future random viral outbreaks. It is emphasized that if used strategically phyto-inhibitors with pre-established clinical data for other diseases can save the time required for long clinical trials. The scientific community should competently tap into phytoconstituents and take their research up to the final stage of clinical trials so that potential phyto-anti-COVID drugs can be developed.

## Introduction

Nobody knew that the coronavirus disease of 2019 (COVID-19) was going to be a greater disaster when compared to the former coronavirus outbreaks, severe acute respiratory syndrome (SARS), and Middle East respiratory syndrome (MERS). The novel coronavirus, severe acute respiratory syndrome coronavirus 2 (SARS-CoV-2), that caused COVID-19 is alarming to the scientific world as it has resulted in the unexpected viral pandemic. However, was COVID-19 an unexpected viral pandemic? The answer is no.

The previous two corona outbreaks, SARS-CoV, and MERS-CoV, were first reported in Guangdong, China in November 2002 (Zhong et al., 2003) and Saudi Arabia in April 2012, respectively (de Wit et al., 2016; Cui et al., 2019). After the identification of SARS-CoV (Ksiazek et al., 2003), several strategies were adopted to eradicate the disease, however, infection control was proven to be more effective than medical intervention leading to the termination of the SARS pandemic. Although the end of the SARS pandemic was announced in July 2003, different types of SARS re-occurred in different years either by zoonotic or human-to-human transmission in several countries including Canada, Vietnam, the Middle East, Hong Kong, South Korea, and Jordan (Wang et al., 2005; Zaki et al., 2012; Hijawi et al., 2013). Later, evidence proved that intermediate hosts may not be required for straight human infection as Chinese horseshoe bats are the main reservoirs of SARS-CoV (Ge et al., 2013). Besides, it was suggested that there was a higher risk of recurrence of SARS-CoV from circulating viruses in bat populations (Menachery et al., 2015).

The emergence of SARS-CoV-2 is just a confirmation of the previous predictions. After previous outbreaks, several studies determined the poor efficacy of therapeutics and both monoclonal antibodies and vaccines against CoV infection (Menachery et al., 2015). In this scenario, new measures should have been created to fight the problem. The fact that more than 619,150 deaths around the world have been reported, as of July 23 2020, has proven that we, as a scientific world, were less prepared for such an abrupt viral outbreak (WHO, 2020, Siuation Report, 185).

Though plants and their extracts have been recognized as effective anti-viral agents for several decades (Chantrill et al., 1952), plant-based medications have been largely ignored. Despite the vast research conducted in several directions after the previous CoV pandemics, plant-based therapeutics could not achieve a satisfactory level in clinical trials against the disease. Medicinal plant extracts have been reported to impede the replication of several viruses including human immunodeficiency virus (HIV), hepatitis B virus (HBV), poxvirus, severe acute respiratory syndrome (SARS) virus, and herpes simplex virus type 2 (HSV-2) (Vermani and Garg, 2002; Kotwal et al., 2005; Huang et al., 2006). Despite that, there are no reports of plant-based medicines that have been successful in preventing the spread of COVID-19 or curing COVID-19. Thus, further study is required to affirm why plant-based medications could not work in the case of COVID-19. In this article, we discuss different aspects of the utilization of plant-based therapeutics in controlling viral outbreaks like COVID-19. Future research for such viral pandemics should be designed in light of the success stories of previous plant-based medications. The disadvantages in this field of research have been deliberated so that lessons can be learned and the scientific community can prepare for future outbreaks.

## Medicinal Plants and Their Extracts as an Anti-Viral Agent

Medicinal plants can be used as anti-viral agents either as first-generation drugs where plant crudes are used in their natural forms or as second-generation drugs where the active metabolites of plants which are responsible for the anti-viral activity are employed (Jassim and Naji, 2003). However, to be successful, plant-based herbal medicines have to address the issue of genetic variability of viruses, competent replication of DNA and RNA viruses within the host cells, and their capability to survive in the host cells (Wagner and Hewlett, 1999; Jassim and Naji, 2003). In contrast to synthetic drugs, some of the plant-based metabolites hinder the replication of viruses without disturbing the host metabolism; which consequently have restricted side effects as drugs (Hussain et al., 2017).

The interest in anti-viral plant research development started with the suppression of the amplification of the influenza A virus by 12 plant extracts (Chantrill et al., 1952). Afterward, continuous efforts have been made to screen different plant sources *via in silico, in vitro*, and *in vivo* assays for anti-viral activity towards several viruses such as parainfluenza virus type 3, respiratory syncytial virus, poliovirus type 1, herpes simplex virus (HSV), enteric coronavirus, and rotavirus (RV). (Ahmad et al., 1996; Rajbhandari et al., 2001; Jassim and Naji, 2003).

Plant crudes contain several metabolites and it is extremely crucial to identify which component makes it a potential candidate for an effective anti-viral drug. Different anti-viral compounds of plants including peptides, lignans, terpenoids, polysaccharides, flavonoids, polyacetylenes, and alkaloids are effective against different targets of viruses such as DNA, RNA genomes, membranes, the replication process, and ribosomal activity (Jassim and Naji, 2003; Ireland et al., 2008; Sencanski et al., 2015; Vilas Boas et al., 2019). As an example, the strong *in vitro* activity of extracts of *Macaranga barteri* against E7 and E19 echoviruses suggests that it can be an effective therapeutic agent for enteroviral infections such as encephalitis (Ogbole et al., 2018). However, among all the components, three stilbenoids (especially vedelianin) isolated from *M. barteri* extracts are largely responsible for the anti-viral activity against echoviruses (Segun et al., 2019). Similarly, other stilbenes such as Resveratrol and trans-arachidin isolated from grapes and the hairy root culture of peanut are capable of reducing the replication rate of the African swine fever virus (ASFV) and RVs, respectively (Abba et al., 2015; Ball et al., 2015).

## Understanding the Coronavirus outbreaks

Coronaviruses, which have the largest genomes among the RNA viruses, consist of a long positive-sense RNA that behaves like mRNA encoding the synthesis of two replicase polyproteins (pp), i.e., pp1a and pp1ab. These polyproteins are processed by a main protease, i.e., chymotrypsin-like (3CLPro) protease and papain like protease (PLP). While the main protease cleaves at 11 sites, PLP cleaves at two or more than two sites on the polyproteins. Due to the essential role of these proteases in proteolytic processing during viral replication, these proteases are considered as the main targets for the development of therapeutic drugs (Deng et al., 2014). CoVs are grouped into four categories: alpha, beta, gamma, and delta types; among which only alpha and beta types are known to infect humans. Coronaviruses are difficult to handle because of the higher mutation rates of their nucleotides as compared to other single-stranded RNA viruses.

Before the present SARS-CoV-2, six different types of human coronaviruses (HCoV) had been discovered including HCoV-HKU1, HCoV-OC43, HCoV-NL63, HCoV-229E, MERS-CoV, and SARS-CoV (Friedman et al., 2018). The enveloped HCoV viruses fit in to Coronaviridae family and are known to develop respiratory diseases (Geller et al., 2012). SARS-CoV and MERS-CoV were reported as the most deadly viral corona outbreaks causing an epidemic in different countries with a fatality rate of 9% (during 2002 and 2003) and 35.4% (up to December 2016), respectively (De Wit et al., 2016).

## The Success Stories of Phytoconstituents Against SARS-CoV and MERS-CoV

The first outbreak of SARS-CoV in China led to a sprint of screening of Chinese medicinal herbs against the disease and some of them came out as potential anti-viral agents. During the SARS outbreak in the absence of effective therapies, ribavirin, a licensed drug for the respiratory syncytial virus (RSV) was commonly suggested as a treatment (Van Vonderen et al., 2003). However, it was later found to cause the death of SARS patients by inducing anemia and hemolysis (Booth et al., 2003; Chiou et al., 2005). Glycyrrhizin (triterpene glycoside glycyrrhizic acid), a phytoconstituent extracted from liquorice roots (*Glycyrrhiza radix*) proved to be a more efficient anti-viral agent for SARS-CoV when compared to ribavirin (Cinatl et al., 2003). Glycyrrhizin was tried against the isolates of SARS-CoV replicated in Vero cells (kidney epithelial cells isolated from African green monkeys) and was proven to be the most compelling interceptor of replication of SARS-CoV when compared to other anti-viral agents such as mycophenolic acid, pyrazofurin, 6-azauridine, and ribavirin (Cinatl et al., 2003). Besides it also hinders the entry of the virus which is a main step in the replication cycle. The concentration of glycyrrhizin required to impede the cytopathic effect of a virus in a Vero cell culture to 50% of the control value, i.e., EC50, was 300 mg/L. EC50 stands for the effective concentration of a drug that gives a half-maximal response. Although the complete effect of glycyrrhizin activity towards SARS-CoV is not clear, it increases the production of nitrous oxide by the overexpression of nitrous oxide synthase that inhibits the viral replication (Jeong and Kim, 2002; Cinatl et al., 2003). A concentration of 1000 mg/L of glycyrrhizin was found to be effective in lowering the expression of SARS-CoV antigens (extracted from patient’s serum) in a Vero cell culture. Further analysis of glycyrrhizin derivatives against SARS-CoV showed that the addition of different compounds to functional groups may lead to a 10-70 fold increase in the anti-SARS activity; however, they may also increase the cytotoxic effects (Hoever et al., 2005). Although cytotoxic effects need to be discussed, such studies open the platform for the modification of glycyrrhizin for the production of novel anti-SARS-CoV drugs with enhanced activity.

Several plants and their extracts have been reported to have a remedial approach against SARS-CoV and MERS-CoV by modulating the immune response. Based on a virus-induced cytopathic effect (CPE) assay and an MTS [(5-(3-carboxymethoxyphenyl)-2-(4,5-dimethyl-thiazoly)-3-(4-sulfophenyl) tetrazolium] cell proliferation assay, extracts of *Linder aggregata*, *Pyrrosia lingua*, *Artemisia annua*, and *Lycoris radiata* with EC50 values ranging from 2.4 ± 0.2 to 88.2 ± 7.7 mg/L showed much better anti-SARS-CoV activity in a Vero cell culture when compared to glycyrrhizin (Li et al., 2005). However, among these four extracts, *Lycoris radiata* with an EC50 value of 2.4 ± 0.2 mg/L was found to be the most effective candidate for anti-viral medicine against SARS-CoV. Lycorine, one of the phytoconstituents fractionated from the extract of *Lycoris radiata* is mainly responsible for its anti-SARS-CoV activity showing a lower EC50 value than its original extract (± 0.0012 µM). Lycorine is also recognized for its potential inhibition activity against herpes simplex virus (type I) and the poliomyelitis virus. Thus, it is crucial to explore the broad anti-viral feature of lycorine in detail using real-time PCR to assess its capacity to inhibit viral RNA replication and its interface with viral antigens. Aescin, an extensively utilized drug in Europe, which is extracted from horse chestnut trees and reserpine which is extracted from the Rauwolfia species have shown EC50 values of 3.4 µM and 6.0 µM against SARS-CoV in a Vero cell culture, respectively (Wu et al., 2004). In addition, *Radix ginseng*, eucalyptus, and *Lonicera japonica* extracts have shown anti-viral activity towards SARS-CoV at 100 µM. Later, Ginsenoside-Rb1 isolated from the traditional Chinese herb, *Radix ginseng*, was reported to lessen acute lung injury in rats by inhibiting the inflammatory signaling pathway (Yuan et al., 2014).

Many research experiments have been conducted to determine the inhibition capacity of phytoconstituents against the main protease (3CLpro) and papain-like protease (PLpro) of SARS and MERS coronaviruses. Strangely, even the constituents in tea can be potentially effective against a deadly virus like SARS-CoV. It is one of the positive points that can be counted on while considering the negative effects of tannic acid in the tea. The 3-isotheaflavin-3-gallate (TF2B), theaflavin-3,3’-digallate (TF3), and tannic acid compounds that are abundant in black tea extracts are potent inhibitors of the main protease of SARS-CoV, 3CLpro at IC50 < 10 µM as determined by an HPLC proteolytic assay (Chen et al., 2005). IC50 stands for the concentration of an inhibitor where the response is reduced by half. Quercetin, a plant flavonoid, and its derivative quercetin-3-β-galactoside show potent inhibition of viral replication in SARS-CoV 3CLpro where sugar moiety is crucial for inhibitory action (Yi et al., 2004; Lin et al., 2005; Chen et al., 2006). An extract of *Houttuynia cordata*, a traditional Chinese medicine, at 200µg/ml had been reported to have an inhibitory effect on the RNA-dependent RNA polymerase (RdRp) and 3C-like protease (3CLpro) of SARS-CoV which was non-toxic to mice at an oral dosage of 16 g/kg (Lau et al., 2008). Scutellarein extracted from *Scutettaria baicalensis* can be a potential SARS-CoV inhibitor as it hinders the ATPase activity of the helicase protein of SARS-CoV *in vitro* (Yu et al., 2012). Few compounds such as dihydrotanshinone isolated from the root of *Salvia miltiorrhiza* showed anti-viral activity against both SARS and MERS CoV by the inhibition of proteases and hindering of the viral entry, respectively (Park et al., 2012; Kim et al., 2018). A phlorotannin, dieckol, extracted from the edible brown algae *Ecklonia cava* showed potential inhibitory effects on the 3CLpro of SARS-CoV at IC50 = 2.7 µM. It is more repressive on the cell-based 3CLpro cis-cleavage when compared to the other natural CoV protease inhibitors such as quinone-methide triterpene extracted from *Tripterygium regelii* (Ryu et al., 2010; Park et al., 2013).

Not only are these extracts potent against 3CLpro but also several natural compounds have been reported to have a greater inhibitory action against PLpro. A polyphenol compound, papyriflavonol A, derived from *Broussonetia papyrifera* which hinders the SARS-CoV PLpro with an IC50 value of 3.7 µM can be utilized for the development of anti-CoV agents (Park et al., 2017). A cinnamic amide with an infrequent carbinolamide motif derived from the methanol extract of *T. terrestris* fruit showed effective inhibitory action towards SARS-CoV PLpro with IC50 = 15.8 µM (Song et al., 2014).

Resveratrol (trans-3, 5, 4′-trihydroxystilbene), a natural stilbene derivative, can be extracted from different plants including cranberry (*Vaccinium macrocarpon*), grape (*Vitis vinifera*), and Huzhang (*Polygonum cuspidatum*) is efficient in inhibiting MERS-CoV replication *in vitro* by reducing cell death and alleviating the expression of nucleocapsid protein that is required for viral replication (Lin et al., 2017). The inhibition of the NF-κB pathway by resveratrol in signal transduction shows its potential capacity to be an effective broad-spectrum anti-viral agent and thus, its functioning against CoV should be investigated *in vivo*.

The anti-viral activity of several medicinal herbal extracts such as *Sophora subprostrata radix*, *Phellodendron cortex*, *Coptidis rhizoma*, *Meliae cortex*, and *Cimicifuga rhizome* was identified against mouse hepatitis virus (MHV) which is widely considered as a prototype of coronavirus. The EC50 values of these compounds is in the range of 2.0 to 27.5 µg/ml suggesting that these can be potent candidates for developing anti-viral therapeutics (Kim et al., 2008).

## Phytoconstituents With Broad-Spectrum Activity Against CoVs

The accidental outbreaks of SARS and MERS coronaviruses pointed towards the chances of the emergence of novel CoVs in the future. Until 2020, there was a dearth of approved drugs for SARS-CoV and MERS-CoV and it emphasized the significance of broad-spectrum viral inhibitors. The extent of conservation in crucial active domains of different human coronaviruses such as RNA helicase and 3CLpro can be utilized as a target when developing potential broad-spectrum anti-CoV drugs. Silvestrol, extracted from *Aglaia* sp., inhibits the cap-dependent mRNA translation of HCoV-229E and MERS-CoV in human embryonic lung fibroblast (MRC-5) cells with EC50 values of less than 0.003 µM and 0.0013 µM, respectively (Muller et al., 2018). Several phytocompounds including mycophenolate mofetil, emetine, and lycorine were identified as the potential broad-spectrum inhibitors that hindered the *in vitro* replication of four CoVs; MERS-CoV, MHV-A59, HCoV-NL63, and HCoV-OC43-WT with EC50 values less than 5 µM (Shen et al., 2019). Among these, the inhibition capacity of lycorine against HCoV-OC43 by reducing the viral lethality in the central nervous system of mice was reported *in vivo via* bioluminescence imaging (Shen et al., 2019). The effectiveness of most of these phytocompounds as broad-spectrum inhibitors has been confirmed in *in vitro* infection models and was tested in a particular cell line that may be influenced by specific host cell types. The identification of broad-spectrum inhibitors and determining their inhibition capacity in an *in vivo* system may lead to the production of potential drugs.

## Where Can We Find Phytoconstituents in the Race of Developing Drugs for Pandemics Like COVID-19?

The emergence of the COVID-19 outbreak and its spread around the world led to an urgent search for a solution against the disease. While expected methods such as the combined medication of systematic corticosteroids and anti-viral treatment along with interferons are being tried for the speedy recovery of patients, plant-based treatment regimes are also potentially being explored in the race. The release of the gene sequence of SARS-CoV-2, the crystallization of its main protease, and its availability in the Protein Data Bank (PDB) showed that the main proteins of SARS-CoV-2 shares great similarities with those of SARS-CoV and MERS-CoV (Zhang L. et al., 2020; Zhou et al., 2020). It should be considered that despite the reported mutations in the novel coronavirus as compared to SARS-CoV and MERS-CoV, the effectiveness of potential plants and their phytoconstituents that were operative against SARS-CoV and MERS-CoV could have been employed for SARS-CoV-2. SARS-CoV and SARS-CoV-2 belong to beta coronaviruses with high homology in the genomic sequence at the nucleotide level; however, there are six regions of differences in their genome sequence. These regions can be supportive to developing new drugs for SARS-CoV-2 (Xu et al., 2020). In addition, proteins of SARS-CoV-2 share 95% - 100% homology with SARS-CoV with only two non-homologous proteins, orf8 and orf10. The amino acid sequence of orf8 is different in both the viruses (Chan et al., 2020). A Blastp comparison of SARS-CoV and SARS-CoV-2 showed a more than 95% similarity in helicase, nsp7,8,9,10, 3C-like proteinase, 3’-to-5’ exonuclease, and RNA-dependent RNA polymerase (Xu et al., 2020). The antibodies active towards the N protein of SARS-CoV may have increased the probability of binding to the N protein of SARS-CoV-2 due to approximately 90% identity in the N protein amino acids of both viruses (Gralinski and Menachery, 2020). These similarities and dissimilarities between the two viruses should be considered while utilizing the phyto-inhibitors active against SARS-CoV for the novel coronavirus (nCoV), SARS-CoV-2. However, not taking potential plants or their phytoconstituents to an effective therapeutic anti-viral drug stage during previous corona outbreaks is one of the major disadvantages of the present scenario. The availability of potent plant-based drugs against SARS-CoV and MERS-CoV could have opened new treatment pathways for sudden outbreaks such as COVID-19.

Given this disadvantage, most of the plant-based investigations directed towards COVID-19 either focus on bioinformatic tools such as *in silico* processing, molecular docking, or concentrate on molecular farming such as the production of recombinant proteins involving vaccines and antibodies (Islam et al., 2020; Rosales-Mendoza et al., 2020). Several molecules of known herbal medicines, when docked with the proteins of SARS-CoV-2, have been reported to inhibit 3CLpro, PLpro, spike proteins, and viral replication by binding in different domains (Islam et al., 2020). This binding hinders the substrate from going to the enzyme’s active sites, prevents dimer formation, or averts viral entry (Park et al., 2013; Zhang D. H. et al., 2020). The traditional herbs which contain these potential anti-viral compounds and are regularly used in handling viral respiratory infections might be employed to provide immediate support in the treatment of COVID-19. However, clinical manifestations are required for the routine implementation of these phytoconstituents as drugs.

**Figure 1:**
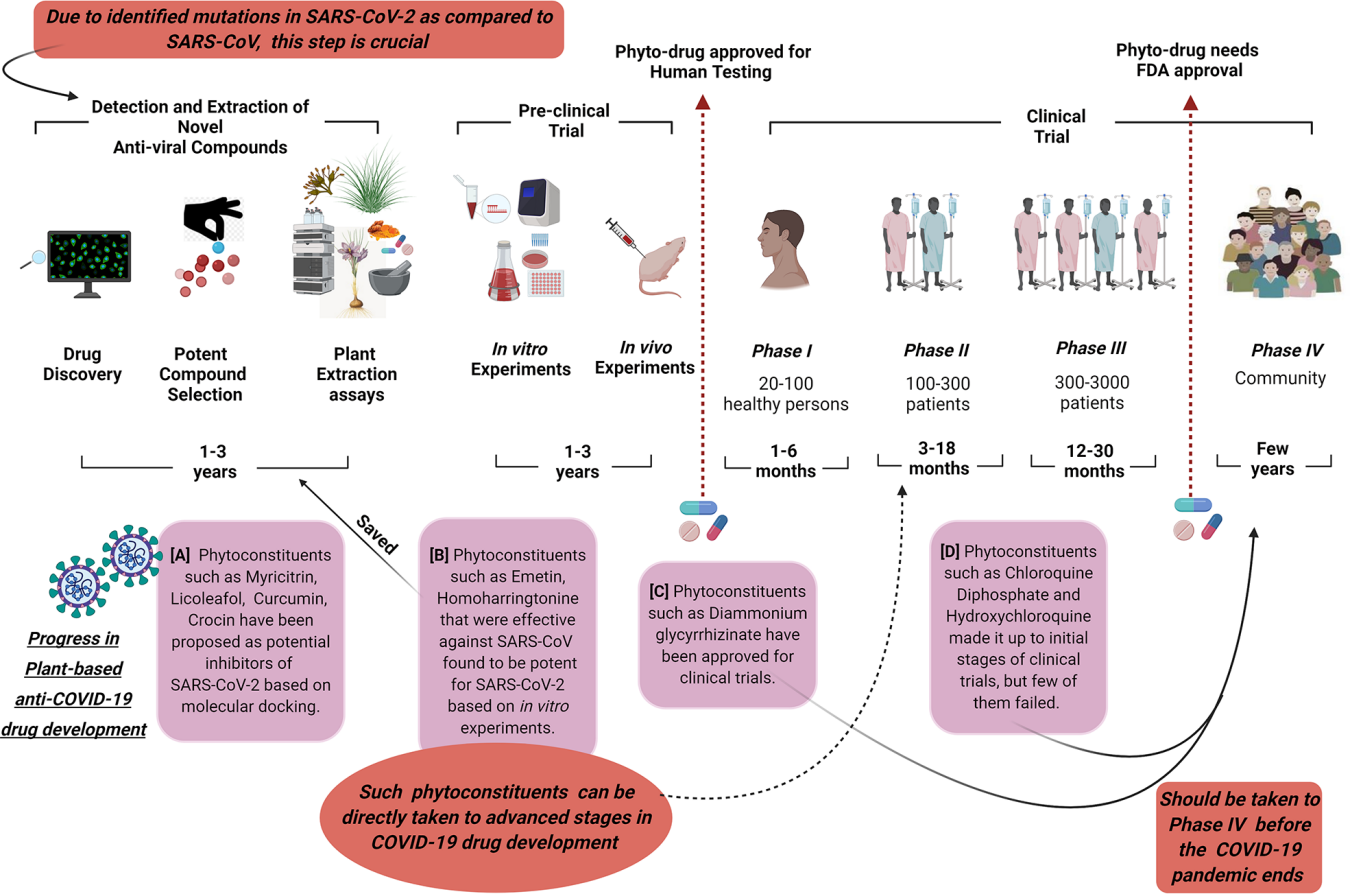
This picture shows the current status of phytoconstituents in the development process of anti-COVID-19 drugs. Due to the identified mutations in SARS-CoV-2 as compared to SARS-CoV, the detection of potent anti-viral compounds is necessary. Molecular docking can largely contribute to this process. The phytocompounds (A) that are identified as potential inhibitors of SARS-CoV-2 should be immediately forwarded to pre-clinical and clinical trials (Aanouz et al., 2020; Khaerunnisa et al., 2020; Ul Qamar et al., 2020). The phytocompounds (B) that were effective against SARS-CoV and are potent for SARS-CoV-2 based on *in vitro* experiments saved the time of target compound selection and extraction assays (Nemunaitis et al., 2013; Choy et al., 2020). As their efficacy and safety has already been proven in previous the SARS-CoV outbreak, these can be directly taken to advanced stages in COVID-19 clinical trials. The phytocompounds (C) that are approved for clinical trials or are currently in the process of clinical trials (D) should be taken forward to phase IV as soon as possible so that the wide effect of the developed drugs can be observed before the COVID-19 pandemic ends (Borba et al., 2020; Gautret et al., 2020; Qiu et al., 2020). This is extremely critical for future random viral corona outbreaks.

Additionally, it should also be kept in mind that molecular docking is a crucial process in the identification of potential anti-viral compounds and is based on the available genome information of the novel coronavirus. In the case of mutation in the existing SARS-CoV-2, the suggested compounds may not be effective and new investigations will be required (**Figure 1**). Ul Qamar et al. (2020) created a 3D homology model of the 3CLpro sequence of SARS-CoV-2 and highlighted its conserved nature comparable with the main protease sequence of SARS-CoV which shared a 99.02% sequence similarity. However, 12 point-mutations have been reported in SARS-CoV-2 which dislocate crucial hydrogen bonds, change the receptor binding site of its main protease, and thus, SARS-CoV-2 may behave differently towards some phyto-inhibitors that were effective towards SARS-CoV and need to be tested. A detailed molecular docking-based screening of more than 32,000 phytoconstituents resulted in nine potential compounds (including myricitrin, licoleafol, and amaranthin) that may hinder the activity of the SARS-CoV-2 3CLpro (Ul Qamar et al., 2020). Another molecular docking-based study using lopinavir and nelfinavir as standards revealed that epicatechin-gallate, catechin, curcumin, oleuropein, apigenin-7-glucoside, naringenin, demethoxycurcumin, luteolin-7-glucoside, quercetin, and kaempferol compounds extracted from medicinal plants may act as potential inhibitors of the main protease of COVID-19 (Khaerunnisa et al., 2020). Three phytocompounds, β-Eudesmol, digitoxigenin, and Ccrocin isolated from *Lauris nobilis* L, *Nerium oleander*, and *Crocus sativus* L, respectively, have been proposed as potential inhibitors of the spike protein of SARS-CoV-2 based on the molecular docking study (Aanouz et al., 2020) (**Figure 1**).

Another important aspect while dealing with phyto-inhibitors against CoVs is that the results obtained from *in vitro* experiments may be different from the clinical efficacy in *in vivo* experiments. This may be due to the fact that the oral intake of these phyto-drugs may not reach the expected blood serum concentration as observed in *in vitro* experiments. Emetine, an alkaloid extracted from the root of the plant *Psychotria ipecacuanha* (ipecac root), that was determined to be a broad-spectrum inhibitor regulating different CoVs *in vitro* was found to inhibit SARS-CoV-2 replication at 0.5 μM. However, as a therapeutic its plasma concentration stretches up to 0.156 μM that is much lower than its toxic plasma concentration of 1.04 μM and its EC50 value towards SARS-CoV-2 (Regenthal et al., 1999; Choy et al., 2020). Homoharringtonine, isolated from *Cephalotoxus fortunei*, is a broad spectrum anti-viral drug effective against murine hepatitis and porcine epidemic diarrhea coronaviruses and inhibits SARS-CoV-2 at EC50 = 2.10 μM. However, a semi-synthetic type of homoharringtonine, omacetaxine, is reported to have a therapeutic plasma concentration of 0.066 μM after 11 days of treatment that was much lower than its EC50 value against SARS-CoV-2 *in vitro* (Nemunaitis et al., 2013; Choy et al., 2020). These results of emetine and homoharringtonine confirmed that plant-based therapeutics which are potentially effective against SARS-CoV-2 should be immediately considered for dose optimization. Isolated from liquorice roots, diammonium glycyrrhizinate combined with Vitamin C tablets had been prescribed for common COVID-19 symptoms and had been approved for randomized clinical trials (Chen et al., 2020; Qiu et al., 2020). These results also give us hope that broad-spectrum phyto-anti-virals can be effective for sudden viral outbreaks in the future.

## Why Were Successful Clinical Trials Not Possible for SARS-CoV Phyto-Inhibitors?

Different researchers are investigating diverse plant forms based on ethnopharmacological data to find effective anti-CoV drugs with novel action mechanisms especially targeting viral replication. In this crucial situation, it is required to discuss why phyto-inhibitors could not reach an effective drug level in previous corona outbreaks so that proper strategies could be developed for future viral epidemics.

The development of drugs is a costly and long-term process. As the screening of plant-based anti-virals is very similar to the testing procedure of synthetic drugs, a better correlation of their *in vitro* and *in vivo* (IVIVC) results may hasten their approval process (Babar, 2013; Bose et al., 2020) (**Figure 1**). The clinical trials of plants-based anti-virals pass through five phases including the pre-clinical phase (unrestricted dose on animals or *in vitro* experiments), phase I, II, III, and IV (**Figure 1**). Phase I includes the testing of the drug and its doses on healthy people, while phase II and phase III are comprised of screening in patients to check the efficacy, side-effects, and safety issues associated with the drug. The number of participants increases in each phase with approximately 300-3000 patients in phase III. Phase IV is one of the most crucial steps including post-marketing surveillance to observe the safety and long-term effects of the drug when used in public. Several potential plant-based anti-virals against different viral diseases have entered into the market of licensed products as they are effective against particular cellular responses without an added destruction of the cell. *Echinacea purpurea* has reached Phase IV of non-randomized clinical trials in Spain to illustrate the interaction between the anti-retroviral drug, darunavir, and *Echinacea purpurea* in HIV-1 infected patients (Mólto et al., 2010; Kurapati et al., 2016). *Triptolide woldifiion* in China made it up to phase III in randomized clinical trials and its impact on the HIV-1 reservoir was estimated (Li, 2014). (+)-Calanolide A extracted from *Calophyllum lanigerum* hinders HIV-1 reverse transcriptase and was one of the few initial anti-HIV agents that went into a clinical trial. It successfully passed the Phase I clinical trial which was performed on healthy people (Creagh et al., 2001); however, it was not further evaluated for efficacy and safety (Usach et al., 2013).

*Phyllanthus urinaria* and *Phyllanthus niruri* were found to block endogenous DNA polymerase enzyme necessary for hepatitis B virus (HBV) replication and made it up to clinical trial (Jassim and Naji, 2003). However, a randomized controlled trial on 47 patients suffering from chronic HBV showed that 12 months of the intervention of 250 mg capsule of *Phyllanthus niruri* twice in a day did not reduce the virus load and could not clear the hepatitis B antigens. Accordingly, *Phyllanthus niruri* was not recommended as a standard drug for chronic hepatitis B patients as its efficiency was found to be wide-ranging according to the variations in the treated populations (Baiguera et al., 2018). Similarly, in the case of COVID-19, two forms of cinchona bark-based antimalarials, chloroquine diphosphate and hydroxychloroquine have also been the center of interest due to the reported inhibitory effects of these compounds on SARS-CoV. Positive reports of recovery in SARS-CoV-2 affected patients after hydroxychloroquine treatment are available where a daily dose of 600mg of hydroxychloroquine along with azithromycin significantly reduced virus load after six days of inclusion (Gautret et al., 2020). However, the main drawback of this study was the small sample size and limited time for a long-term follow-up of the patients. Another study on chloroquine diphosphate reported a lethality rate of 15% and 39% in low-dosage (450 mg twice a day on the 1st day and once a day for 4 days) and high-dosage (600 mg twice a day for 10 days) groups in critical SARS-CoV-2 patients after 13 days of treatment (Borba et al., 2020). Thus, a higher dosage of chloroquine diphosphate along with azithromycin in critically ill patients especially suffering from cardio disorders has been reported to be unsafe. Thus, evaluation of chloroquine as a drug through randomized clinical trials is required. The chances of the effective use of similar phytocompounds in the COVID-19 outbreak would have been higher if experiments focusing on these phyto-inhibitors had been performed for and after SARS-CoV.

One of the possible reasons for the failure of such natural products could be the differences in the prepared drug due to the ecological and seasonal variations in the plant growth, genotypic variation, timing of harvest, variations in the storage, and manufacturing conditions (Islam et al., 2020). Changes in these factors may influence the production of the main component and contaminants (Shimanovskii, 2020). Studies such as that of Borba et al. (2020) and Gautret et al. (2020) reported a daily requirement of a minimum 600 mg of chloroquine compounds per SARS-CoV-2 patient. Though plants produce enough of these natural products for their own use, it is not sufficient to fulfill the commercial manufacturing needs of pharmaceutical companies. Thus, sustainable and reproducible large-scale production of these natural products is another challenge in their successful utilization for the treatment of SARS-CoV-2. Plant cell and tissue culture approaches can be effective for the extraction and multiplication of many of these natural constituents (Hussain et al., 2012; Shimanovskii, 2020). Extraction of phytocompounds from the tissue culture is quick and efficient when compared to the isolation from whole plants. Moreover, plant tissue culture techniques facilitate the production of phytocompounds in completely controlled conditions following the regulations of good manufacturing practices (GMP). Phytocompounds extracted from tissue cultures can be free of microbes and other compounds found in soil-grown plants and are protected from climatic changes (Hussain et al., 2012). Other than plant tissue culture methods, vertical farming units (VFUs) can be promising for the controlled and monitored exponential production of the target crop that prevents the cross-pollination of the target crop with genetically compatible species (Ma et al., 2005; Buyel et al., 2017; Buyel, 2018). Not only for growing and harvesting the plants, GMPs must be followed for the extraction and purification of the pure and homogenous phytocompound from the harvested biomass. This downstream processing of phytocompounds may account for approximately 80% of the total production cost depending on the removal of the contaminants and purity of the extracted compound (Ma et al., 2005; Fischer et al., 2012). Moreover, the chances of success of natural anti-viral products may largely increase if they are prepared in line with ethnopharmacological guidelines. The proper application of in silico and *in vitro* methods followed by *in vivo* experiments may smooth the way for clinical trials of phytocompounds. More than a hundred phytocompounds have been found to inhibit different types of coronaviruses either by inhibiting the interaction of the SARS-CoV (S) protein and the ACE2 receptor or by inhibiting the viral replication, cell division, 3CL protease, papain-like protease (PL pro) or by hindering the viral entry (Islam et al., 2020). It should be noted that for SARS-CoV that emerged in 2003, it was not until 2017 when *in vitro* studies confirming the action mechanism of natural products were conducted (Kim et al., 2014; Schwarz et al., 2014; Park et al., 2017); however, the natural products with the same mechanism against SARS-CoV and even more effective mechanisms were already identified in the initial years of the SARS-CoV epidemic. If more efforts had been given to those phyto-resources from the start of the SARS-CoV epidemic, they could have reached successful clinical trials. Moreover, the inability of SARS-CoV phyto-inhibitors-based research to reach an extensive level of *in vivo* studies reduced the chances of phyto-anti-CoV drugs being developed and passing successfully through clinical trials.

## Conclusion

The emergency SARS-CoV-2 outbreak led to the utilization of several phytocompounds for the treatment of patients. The direct benefit of most of these compounds is that their effects on the human body and their safety are already established through clinical trials for other diseases. However, utmost care is required while prescribing the dose of these phytocompounds as their uncontrolled use can have long term side-effects on the patient’s body. Though numerous phytoconstituents were found to be effective against SARS and MERS CoVs, they fell behind in the drug development process as most of them could not reach the clinical trial stage. One of the possible reasons for incomplete clinical trials could be the intermittent nature of the SARS and MERS epidemics. The absence of patients for the advanced phase trials (phase II, III, IV) of phytoconstituents may have contributed to their failure as licensed drugs. Thus, one of the potential suggestions for COVID-19 recovery could be to identify the potential phytoconstituents based on *in vitro* results and with minimum side effects in phase I trials and take them up to advanced phase trials (phase II, III, IV) as soon as possible (**Figure 1**). So that, in case the COVID-19 pandemic ends abruptly by chance like SARS-CoV, then we can have at least a few efficient phyto-anti-COVID drugs that would have completed randomized clinical trials. Such drugs may not only be effective on re-emergence of SARS-CoV-2 in the coming years but can also be potent against similar viral respiratory outbreaks. Moreover, creating an effective phyto-anti-COVID drug during this pandemic may provide an idea on the duration and the strategy required for the development of potent plant-based therapeutics in case of such random viral outbreaks (**Figure 1**). We as plant biologists need to be more concerned and vigilant about the status of the plants, their extracts, and phytoconstituents in developing phyto-anti-viral drugs and controlling pandemics like COVID-19. It is as crucial as the production of important cereals in the world.

## Author Contributions

AP and MKK conceived, wrote, and edited the manuscript. MH and SG made intellectual contributions to the manuscript. All authors contributed to the article and approved the submitted version.

## Conflict of Interest

The authors declare that the research was conducted in the absence of any commercial or financial relationships that could be construed as a potential conflict of interest.

## References

[B1] Aanouz I. Belhassan A. El Khatabi K. Lakhlifi T. El Idrissi M. Bouachrine M. (2020). Moroccan Medicinal plants as inhibitors of COVID-19: Computational investigations. J. Biomol. Struct. Dynamics 38, 1–12. 10.1080/07391102.2020.1758790 PMC7212546 32306860

[B2] Abba Y. Hassim H. Hamzah H. Noordin M. M. (2015). Antiviral activity of resveratrol against human and animal viruses. Adv. Virol. 2015. 10.1155/2015/184241 PMC4676993 26693226

[B3] Ahmad A. Davies J. Randall S. Skinner G. (1996). Antiviral properties of extract of Opuntia streptacantha. Antiviral Res. 30, 75–85. 10.1016/0166-3542(95)00839-X 8783800

[B4] Babar M. (2013). Antiviral Drug Therapy- Exploiting Medicinal Plants. J. Antivirals Antiretrovirals 05, 28–36. 10.4172/jaa.1000060

[B5] Baiguera C. Boschetti A. Raffetti E. Zanini B. Puoti M. Donato F. (2018). Phyllanthus niruri versus Placebo for Chronic Hepatitis B Virus Infection: A Randomized Controlled Trial. Complement. Med. Res. 25, 376–382. 10.1159/000484927 30372693

[B6] Ball J. M. Medina-Bolivar F. Defrates K. Hambleton E. Hurlburt M. E. Fang L. (2015). Investigation of stilbenoids as potential therapeutic agents for rotavirus gastroenteritis. Adv. Virol. 2015. 10.1155/2015/293524 PMC4563088 26379708

[B7] Booth C. M. Matukas L. M. Tomlinson G. A. Rachlis A. R. Rose D. B. Dwosh H. A. (2003). Clinical features and short-term outcomes of 144 patients with SARS in the greater Toronto area. Jama 289, 2801–2809. 10.1001/jama.289.21.JOC30885 12734147

[B8] Borba M. G. S. Val F. F. A. Sampaio V. S. Alexandre M. A. A. Melo G. C. Brito M. (2020). Effect of High vs Low Doses of Chloroquine Diphosphate as Adjunctive Therapy for Patients Hospitalized With Severe Acute Respiratory Syndrome Coronavirus 2 (SARS-CoV-2) Infection. JAMA Netw. Open 3, e208857. 10.1001/jamanetworkopen.2020.8857 32330277 PMC12124691

[B9] Bose S. Malik J. Mandal S. C. (2020). “Application of Phytochemicals in Pharmaceuticals,” in Advances in Pharmaceutical Biotechnology, vol. 55-68 (Singapore: Springer).

[B10] Buyel J. F. Twyman R. M. Fischer R. (2017). Very-large-scale production of antibodies in plants: The biologization of manufacturing. Biotechnol. Adv. 35, 458–465. 10.1016/j.biotechadv.2017.03.011 28347720

[B11] Buyel J. F. (2018). Plants as sources of natural and recombinant anti-cancer agents. Biotechnol. Adv. 36, 506–520. 10.1016/j.biotechadv.2018.02.002 29408560

[B12] Chan J. F.-W. Kok K.-H. Zhu Z. Chu H. To K. K.-W. Yuan S. (2020). Genomic characterization of the 2019 novel human-pathogenic coronavirus isolated from a patient with atypical pneumonia after visiting Wuhan. Emerg. Microbes Infect. 9, 221–236. 10.1080/22221751.2020.1719902 31987001 PMC7067204

[B13] Chantrill B. Coulthard C. Dickinson L. Inkley G. Morris W. Pyle A. (1952). The action of plant extracts on a bacteriophage of Pseudomonas pyocyanea and on influenza A virus. Microbiology 6, 74–84. 10.1099/00221287-6-1-2-74 14927853

[B14] Chen C. N. Lin C. P. Huang K. K. Chen W. C. Hsieh H. P. Liang P. H. (2005). Inhibition of SARS-CoV 3C-like Protease Activity by Theaflavin-3,3’-digallate (TF3). Evid. Based. Complement Alternat. Med. 2, 209–215. 10.1093/ecam/neh081 15937562 PMC1142193

[B15] Chen L. Li J. Luo C. Liu H. Xu W. Chen G. (2006). Binding interaction of quercetin-3-β-galactoside and its synthetic derivatives with SARS-CoV 3CLpro: Structure–activity relationship studies reveal salient pharmacophore features. Bioorg. Med. Chem. 14, 8295–8306. 10.1016/j.bmc.2006.09.014 17046271 PMC7125754

[B16] Chen L. Hu C. Hood M. Zhang X. Zhang L. Kan J. (2020). A Novel Combination of Vitamin C, Curcumin and Glycyrrhizic Acid Potentially Regulates Immune and Inflammatory Response Associated with Coronavirus Infections: A Perspective from System Biology Analysis. Nutrients 12, 1193. 10.3390/nu12041193 PMC7230237 32344708

[B17] Chiou H. E. Liu C. L. Buttrey M. J. Kuo H. P. Liu H. W. Kuo H. T. (2005). Adverse effects of ribavirin and outcome in severe acute respiratory syndrome: experience in two medical centers. Chest 128, 263–272. 10.1378/chest.128.1.263 16002945 PMC7094379

[B18] Choy K. T. Wong A. Y. Kaewpreedee P. Sia S. F. Chen D. Hui K. P. Y. (2020). Remdesivir, lopinavir, emetine, and homoharringtonine inhibit SARS-CoV-2 replication in vitro. Antiviral Res. 178, 104786. 10.1016/j.antiviral.2020.104786 32251767 PMC7127386

[B19] Cinatl J. Morgenstern B. Bauer G. Chandra P. Rabenau H. Doerr H. W. (2003). Glycyrrhizin, an active component of liquorice roots, and replication of SARS-associated coronavirus. Lancet 361, 2045–2046. 10.1016/S0140-6736(03)13615-X 12814717 PMC7112442

[B20] Creagh T. Ruckle J. L. Tolbert D. T. Giltner J. Eiznhamer D. A. Dutta B. (2001). Safety and pharmacokinetics of single doses of (+)-calanolide a, a novel, naturally occurring nonnucleoside reverse transcriptase inhibitor, in healthy, human immunodeficiency virus-negative human subjects. Antimicrobial Agents Chemother. 45, 1379–1386. 10.1128/AAC.45.5.1379-1386.2001 PMC90477 11302799

[B21] Cui J. Li F. Shi Z.-L. (2019). Origin and evolution of pathogenic coronaviruses. Nat. Rev. Microbiol. 17, 181–192. 10.1038/s41579-018-0118-9 30531947 PMC7097006

[B22] De Wit E. Van Doremalen N. Falzarano D. Munster V. J. (2016). SARS and MERS: recent insights into emerging coronaviruses. Nat. Rev. Microbiol. 14, 523–534. 10.1038/nrmicro.2016.81 27344959 PMC7097822

[B23] Deng X. Stjohn S. E. Osswald H. L. O’brien A. Banach B. S. Sleeman K. (2014). Coronaviruses resistant to a 3C-like protease inhibitor are attenuated for replication and pathogenesis, revealing a low genetic barrier but high fitness cost of resistance. J. Virol. 88, 11886–11898. 10.1128/JVI.01528-14 25100843 PMC4178758

[B24] Fischer R. Schillberg S. Hellwig S. Twyman R. M. Drossard J. (2012). GMP issues for recombinant plant-derived pharmaceutical proteins. Biotechnol. Adv. 30, 434–439. 10.1016/j.biotechadv.2011.08.007 21856403

[B25] Friedman N. Alter H. Hindiyeh M. Mendelson E. Shemer Avni Y. Mandelboim M. (2018). Human Coronavirus Infections in Israel: Epidemiology, Clinical Symptoms and Summer Seasonality of HCoV-HKU1. Viruses 10, 515 10.3390/v10100515 PMC6213580 30241410

[B26] Gautret P. Lagier J. C. Parola P. Hoang V. T. Meddeb L. Mailhe M. (2020). Hydroxychloroquine and azithromycin as a treatment of COVID-19: results of an open-label non-randomized clinical trial. Int. J. Antimicrob. Agents 56. 10.1016/j.ijantimicag.2020.105949 PMC7102549 32205204

[B27] Ge X.-Y. Li J.-L. Yang X.-L. Chmura A. A. Zhu G. Epstein J. H. (2013). Isolation and characterization of a bat SARS-like coronavirus that uses the ACE2 receptor. Nature 503, 535–538. 10.1038/nature12711 24172901 PMC5389864

[B28] Geller C. Varbanov M. Duval R. E. (2012). Human coronaviruses: insights into environmental resistance and its influence on the development of new antiseptic strategies. Viruses 4, 3044–3068. 10.3390/v4113044 23202515 PMC3509683

[B29] Gralinski L. E. Menachery V. D. (2020). Return of the Coronavirus: 2019-nCoV. Viruses 12, 135. 10.3390/v12020135 PMC7077245 31991541

[B30] Hijawi B. Abdallat M. Sayaydeh A. Alqasrawi S. Haddadin A. Jaarour N. (2013). Novel coronavirus infections in Jordan, April 2012: epidemiological findings from a retrospective investigation. EMHJ-Eastern Mediterranean Health J. 19 (Supp. 1) S12-S18 2013. 19, 12–18. 10.26719/2013.19.supp1.S12 23888790

[B31] Hoever G. Baltina L. Michaelis M. Kondratenko R. Baltina L. Tolstikov G. A. (2005). Antiviral Activity of Glycyrrhizic Acid Derivatives against SARS– Coronavirus. J. Med. Chem. 48, 1256–1259. 10.1021/jm0493008 15715493

[B32] Huang K.-L. Lai Y.-K. Lin C.-C. Chang J.-M. (2006). Inhibition of hepatitis B virus production by Boehmeria nivea root extract in HepG2 2.2. 15 cells. World J. Gastroenterol.: WJG 12, 5721–5725. 10.3748/wjg.v12.i35.5721 17007029 PMC4088177

[B33] Hussain M. S. Fareed S. Saba Ansari M. Rahman A. Ahmad I. Z. Saeed M. (2012). Current approaches toward production of secondary plant metabolites. J. Pharm. Bioallied Sci. 4, 10. 10.4103/0975-7406.92725 22368394 PMC3283951

[B34] Hussain W. Haleem K. S. Khan I. Tauseef I. Qayyum S. Ahmed B. (2017). Medicinal plants: a repository of antiviral metabolites. Future Virol. 12, 299–308. 10.2217/fvl-2016-0110

[B35] Ireland D. C. Wang C. K. Wilson J. A. Gustafson K. R. Craik D. J. (2008). Cyclotides as natural anti-HIV agents. Pept. Sci. 90, 51–60. 10.1002/bip.20886 PMC6296364 18008336

[B36] Islam M. T. Sarkar C. El-Kersh D. M. Jamaddar S. Uddin S. J. Shilpi J. A. (2020). Natural products and their derivatives against coronavirus: A review of the non-clinical and pre-clinical data. Phytother. Res. 2020, 1–22. 10.1002/ptr.6700 32248575

[B37] Jassim S. A. Naji M. A. (2003). Novel antiviral agents: a medicinal plant perspective. J. Appl. Microbiol. 95, 412–427. 10.1046/j.1365-2672.2003.02026.x 12911688

[B38] Jeong H. G. Kim J. Y. (2002). Induction of inducible nitric oxide synthase expression by 18β-glycyrrhetinic acid in macrophages. FEBS Lett. 513, 208–212. 10.1016/S0014-5793(02)02311-6 11904152

[B39] Khaerunnisa S. Kurniawan H. Awaluddin R. Suhartati S. Soetjipto S. (2020). Potential Inhibitor of COVID-19 Main Protease (M^pro^) From Several Medicinal Plant Compounds by Molecular Docking Study. Preprints, 2020030226. 10.20944/preprints202003.0226.v1

[B40] Kim H. Y. Shin H. S. Park H. Kim Y. C. Yun Y. G. Park S. (2008). In vitro inhibition of coronavirus replications by the traditionally used medicinal herbal extracts, Cimicifuga rhizoma, Meliae cortex, Coptidis rhizoma, and Phellodendron cortex. J. Clin. Virol. 41, 122–128. 10.1016/j.jcv.2007.10.011 18036887 PMC7108295

[B41] Kim D. W. Seo K. H. Curtis-Long M. J. Oh K. Y. Oh J.-W. Cho J. K. (2014). Phenolic phytochemical displaying SARS-CoV papain-like protease inhibition from the seeds of Psoralea corylifolia. J. Enzyme Inhibition Med. Chem. 29, 59–63. 10.3109/14756366.2012.753591 23323951

[B42] Kim J. Y. Kim Y. I. Park S. J. Kim I. K. Choi Y. K. Kim S.-H. (2018). Safe, high-throughput screening of natural compounds of MERS-CoV entry inhibitors using a pseudovirus expressing MERS-CoV spike protein. Int. J. Antimicrobial Agents 52, 730–732. 10.1016/j.ijantimicag.2018.05.003 PMC7125825 29772395

[B43] Kotwal G. J. Kaczmarek J. N. Leivers S. Ghebremariam Y. T. Kulkarni A. P. Bauer G. (2005). Anti-HIV, Anti-Poxvirus, and Anti-SARS Activity of a Nontoxic, Acidic Plant Extract from the Trifollium Species Secomet-V/anti-Vac Suggests That It Contains a Novel Broad-Spectrum Antiviral. Ann. New Y. Acad. Sci. 1056, 293. 10.1196/annals.1352.014 PMC7167892 16387696

[B44] Ksiazek T. G. Erdman D. Goldsmith C. S. Zaki S. R. Peret T. Emery S. (2003). A Novel Coronavirus Associated with Severe Acute Respiratory Syndrome. New Engl. J. Med. 348, 1953–1966. 10.1056/NEJMoa030781 12690092

[B45] Kurapati K. R. V. Atluri V. S. Samikkannu T. Garcia G. Nair M. P. N. (2016). Natural Products as Anti-HIV Agents and Role in HIV-Associated Neurocognitive Disorders (HAND): A Brief Overview. Front. Microbiol. 6, 1444. 10.3389/fmicb.2015.01444 26793166 PMC4709506

[B46] Lau K. M. Lee K. M. Koon C. M. Cheung C. S. Lau C. P. Ho H. M. (2008). Immunomodulatory and anti-SARS activities of Houttuynia cordata. J. Ethnopharmacol. 118, 79–85. 10.1016/j.jep.2008.03.018 18479853 PMC7126383

[B47] Li T (2014). Clinicaltrials.gov, Study on the Impact of Triptolide Woldifiion on HIV-1 Reservoir In Acute HIV-1 Infection. Available at: Clinicaltrials.gov.

[B48] Li S. Y. Chen C. Zhang H. Q. Guo H. Y. Wang H. Wang L. (2005). Identification of natural compounds with antiviral activities against SARS-associated coronavirus. Antiviral Res. 67, 18–23. 10.1016/j.antiviral.2005.02.007 15885816 PMC7114104

[B49] Lin C.-W. Tsai F.-J. Tsai C.-H. Lai C.-C. Wan L. Ho T.-Y. (2005). Anti-SARS coronavirus 3C-like protease effects of Isatis indigotica root and plant-derived phenolic compounds. Antiviral Res. 68, 36–42. 10.1016/j.antiviral.2005.07.002 16115693 PMC7114321

[B50] Lin S. C. Ho C. T. Chuo W. H. Li S. Wang T. T. Lin C. C. (2017). Effective inhibition of MERS-CoV infection by resveratrol. BMC Infect. Dis. 17, 144. 10.1186/s12879-017-2253-8 28193191 PMC5307780

[B51] Ma J. K. C. Chikwamba R. Sparrow P. Fischer R. Mahoney R. Twyman R. M. (2005). Plant-derived pharmaceuticals – the road forward. Trends Plant Sci. 10, 580–585. 10.1016/j.tplants.2005.10.009 16290220

[B52] Menachery V. D. Yount B. L. Debbink K. Agnihothram S. Gralinski L. E. Plante J. A. (2015). A SARS-like cluster of circulating bat coronaviruses shows potential for human emergence. Nat. Med. 21, 1508–1513. 10.1038/nm.3985 26552008 PMC4797993

[B53] Moltó J. Valle M Miranda C. Cedeño S. Negredo E. Barbanoj MJ. (2011). Herb-Drug Interaction between *Echinacea purpurea* and Darunavir-Ritonavir in HIV-Infected Patients. Antimicrob. Agents Chemother. 55, 326. 21078942 10.1128/AAC.01082-10 PMC3019656

[B54] Muller C. Schulte F. W. Lange-Grunweller K. Obermann W. Madhugiri R. Pleschka S. (2018). Broad-spectrum antiviral activity of the eIF4A inhibitor silvestrol against corona- and picornaviruses. Antiviral Res. 150, 123–129. 10.1016/j.antiviral.2017.12.010 29258862 PMC7113723

[B55] Nemunaitis J. Mita A. Stephenson J. Mita M. M. Sarantopoulos J. Padmanabhan-Iyer S. (2013). Pharmacokinetic study of omacetaxine mepesuccinate administered subcutaneously to patients with advanced solid and hematologic tumors. Cancer Chemother. Pharmacol. 71, 35–41. 10.1007/s00280-012-1963-2 23053254 PMC3535355

[B56] Ogbole O. O. Akinleye T. E. Segun P. A. Faleye T. C. Adeniji A. J. (2018). In vitro antiviral activity of twenty-seven medicinal plant extracts from Southwest Nigeria against three serotypes of echoviruses. Virol. J. 15, 110. 10.1186/s12985-018-1022-7 30021589 PMC6052623

[B57] Park J.-Y. Kim J. H. Kim Y. M. Jeong H. J. Kim D. W. Park K. H. (2012). Tanshinones as selective and slow-binding inhibitors for SARS-CoV cysteine proteases. Bioorg. Med. Chem. 20, 5928–5935. 10.1016/j.bmc.2012.07.038 22884354 PMC7127169

[B58] Park J. Y. Kim J. H. Kwon J. M. Kwon H. J. Jeong H. J. Kim Y. M. (2013). Dieckol, a SARS-CoV 3CL(pro) inhibitor, isolated from the edible brown algae Ecklonia cava. Bioorg. Med. Chem. 21, 3730–3737. 10.1016/j.bmc.2013.04.026 23647823 PMC7126891

[B59] Park J.-Y. Yuk H. J. Ryu H. W. Lim S. H. Kim K. S. Park K. H. (2017). Evaluation of polyphenols from Broussonetia papyrifera as coronavirus protease inhibitors. J. Enzyme Inhibition Med. Chem. 32, 504–512. 10.1080/14756366.2016.1265519 PMC6010046 28112000

[B60] Qiu R. Wei X. Zhao M. Zhong C. Zhao C. Hu J. (2020). Outcome reporting from protocols of clinical trials of Coronavirus Disease 2019 (COVID-19): a review. medRxiv 2020. 10.1101/2020.03.04.20031401

[B61] Rajbhandari M. Wegner U. Jülich M. Schoepke T. Mentel R. (2001). Screening of Nepalese medicinal plants for antiviral activity. J. Ethnopharmacol. 74, 251–255. 10.1016/S0378-8741(00)00374-3 11274826

[B62] Regenthal R. Krueger M. Koeppel C. Preiss R. (1999). Drug levels: therapeutic and toxic serum/plasma concentrations of common drugs. J. Clin. Monitor. Comput. 15, 529–544. 10.1023/A:1009935116877 12578052

[B63] Rosales-Mendoza S. Márquez-Escobar V. A. González-Ortega O. Nieto-Gómez R. Arévalo-Villalobos J. I. (2020). What Does Plant-Based Vaccine Technology Offer to the Fight against COVID-19? Vaccines 8, 183. 10.3390/vaccines8020183 PMC7349371 32295153

[B64] Ryu Y. B. Jeong H. J. Kim J. H. Kim Y. M. Park J.-Y. Kim D. (2010). Biflavonoids from Torreya nucifera displaying SARS-CoV 3CLpro inhibition. Bioorg. Med. Chem. 18, 7940–7947. 10.1016/j.bmc.2010.09.035 20934345 PMC7126309

[B65] Schwarz S. Sauter D. Wang K. Zhang R. Sun B. Karioti A. (2014). Kaempferol derivatives as antiviral drugs against the 3a channel protein of coronavirus. Planta Med. 80, 177–182. 10.1055/s-0033-1360277 24458263 PMC7171712

[B66] Segun P. A. Ogbole O. O. Akinleye T. E. Faleye T. O. C. Adeniji A. J. (2019). In vitro anti-enteroviral activity of stilbenoids isolated from the leaves of Macaranga barteri. Nat. Prod. Res. 37, 1–5. 10.1080/14786419.2019.1644505 31343270

[B67] Sencanski M. Radosevic D. Perovic V. Gemovic B. Stanojevic M. Veljkovic N. (2015). Natural products as promising therapeutics for treatment of influenza disease. Curr. Pharm. Design 21, 5573–5588. 10.2174/1381612821666151002113426 26429712

[B68] Shen L. Niu J. Wang C. Huang B. Wang W. Zhu N. (2019). High-throughput screening and identification of potent broad-spectrum inhibitors of coronaviruses. J. Virol. 93, e00023–e00019. 10.1128/JVI.00023-19 30918074 PMC6613765

[B69] Shimanovskii N. (2020). The Role of Phytoengineering in the Preparation and Production of Herbal Medicines. Pharm. Chem. J. 53, 961–963. 10.1007/s11094-020-02105-1

[B70] Song Y. H. Kim D. W. Curtis-Long M. J. Yuk H. J. Wang Y. Zhuang N. (2014). Papain-like protease (PLpro) inhibitory effects of cinnamic amides from Tribulus terrestris fruits. Biol. Pharm. Bull. 37, 1021–1028. 10.1248/bpb.b14-00026 24882413

[B71] Ul Qamar M. T. Alqahtani S. M. Alamri M. A. Chen L. L. (2020). Structural basis of SARS-CoV-2 3CL(pro) and anti-COVID-19 drug discovery from medicinal plants. J. Pharm. Anal. 10, 313–319. 10.1016/j.jpha.2020.03.009 32296570 PMC7156227

[B72] Usach I. Melis V. Peris J. E. (2013). Non-nucleoside reverse transcriptase inhibitors: a review on pharmacokinetics, pharmacodynamics, safety and tolerability. J. Int. AIDS Soc. 16, 1–14. 10.7448/IAS.16.1.18567 24008177 PMC3764307

[B73] Van Vonderen M. G. Bos J. C. Prins J. M. Wertheim-Van Dillen P. Speelman P. (2003). Ribavirin in the treatment of severe acute respiratory syndrome (SARS). Neth J. Med. 61, 238–241. 14567520

[B74] Vermani K. Garg S. (2002). Herbal medicines for sexually transmitted diseases and AIDS. J. Ethnopharmacol. 80, 49–66. 10.1016/S0378-8741(02)00009-0 11891087

[B75] Vilas Boas L. C. P. Campos M. L. Berlanda R. L. A. De Carvalho Neves N. Franco O. L. (2019). Antiviral peptides as promising therapeutic drugs. Cell Mol. Life Sci. 76, 3525–3542. 10.1007/s00018-019-03138-w 31101936 PMC7079787

[B76] Wagner E. Hewlett M. (1999). Basic virology (p. 656). Malden MA: Blackwell Sci.

[B77] Wang M. Yan M. Xu H. Liang W. Kan B. Zheng B. (2005). SARS-CoV infection in a restaurant from palm civet. Emerg. Infect. Dis. 11, 1860. 10.3201/eid1112.041293 16485471 PMC3367621

[B78] World Health Organization (2020). Coronavirus disease 2019 (COVID-19): situation report, 185. World Health Organization Available at: https://apps.who.int/iris/handle/10665/333573

[B79] Wu C. Y. Jan J. T. Ma S. H. Kuo C. J. Juan H. F. Cheng Y. S. (2004). Small molecules targeting severe acute respiratory syndrome human coronavirus. Proc. Natl. Acad. Sci. U.S.A. 101, 10012–10017. 10.1073/pnas.0403596101 15226499 PMC454157

[B80] Xu J. Zhao S. Teng T. Abdalla A. E. Zhu W. Xie L. (2020). Systematic Comparison of Two Animal-to-Human Transmitted Human Coronaviruses: SARS-CoV-2 and SARS-CoV. Viruses 12, 244. 10.3390/v12020244 PMC7077191 32098422

[B81] Yi L. Li Z. Yuan K. Qu X. Chen J. Wang G. (2004). Small molecules blocking the entry of severe acute respiratory syndrome coronavirus into host cells. J. Virol. 78, 11334–11339. 10.1128/JVI.78.20.11334-11339.2004 15452254 PMC521800

[B82] Yu M.-S. Lee J. Lee J. M. Kim Y. Chin Y.-W. Jee J.-G. (2012). Identification of myricetin and scutellarein as novel chemical inhibitors of the SARS coronavirus helicase, nsP13. Bioorg. Med. Chem. Lett. 22, 4049–4054. 10.1016/j.bmcl.2012.04.081 22578462 PMC7127438

[B83] Yuan Q. Jiang Y. W. Ma T. T. Fang Q. H. Pan L. (2014). Attenuating effect of Ginsenoside Rb1 on LPS-induced lung injury in rats. J. Inflammation (Lond) 11, 40. 10.1186/s12950-014-0040-5 PMC4272525 25530718

[B84] Zaki A. M. Van Boheemen S. Bestebroer T. M. Osterhaus A. D. Fouchier R. A. (2012). Isolation of a novel coronavirus from a man with pneumonia in Saudi Arabia. New Engl. J. Med. 367, 1814–1820. 10.1056/NEJMoa1211721 23075143

[B85] Zhang D. H. Wu K. L. Zhang X. Deng S. Q. Peng B. (2020). In silico screening of Chinese herbal medicines with the potential to directly inhibit 2019 novel coronavirus. J. Integr. Med. 18, 152–158. 10.1016/j.joim.2020.02.005 32113846 PMC7102521

[B86] Zhang L. Lin D. Sun X. Curth U. Drosten C. Sauerhering L. (2020). Crystal structure of SARS-CoV-2 main protease provides a basis for design of improved α-ketoamide inhibitors. Science 368, 409–412. 10.1126/science.abb3405 32198291 PMC7164518

[B87] Zhong N. S. Zheng B. J. Li Y. M. Poon L. L. M. Xie Z. H. Chan K. H. (2003). Epidemiology and cause of severe acute respiratory syndrome (SARS) in Guangdong, People’s Republic of China, in February 2003. Lancet 362, 1353–1358. 10.1016/S0140-6736(03)14630-2 14585636 PMC7112415

[B88] Zhou P. Yang X.-L. Wang X.-G. Hu B. Zhang L. Zhang W. (2020). A pneumonia outbreak associated with a new coronavirus of probable bat origin. Nature 579, 270–273. 10.1038/s41586-020-2012-7 32015507 PMC7095418

